# Expression and Subcellular Distribution of GFP-Tagged Human Tetraspanin Proteins in *Saccharomyces cerevisiae*

**DOI:** 10.1371/journal.pone.0134041

**Published:** 2015-07-28

**Authors:** Karin Skaar, Henryk J. Korza, Michael Tarry, Petra Sekyrova, Martin Högbom

**Affiliations:** 1 Department of Biochemistry and Biophysics, Stockholm University, Stockholm, Sweden; 2 Department of Pharmacology and Physiology, Karolinska Institutet, Stockholm, Sweden; 3 Central European Institute of Technology, Masaryk University, Brno, Czech Republic; Griffith University, AUSTRALIA

## Abstract

Tetraspanins are integral membrane proteins that function as organizers of multimolecular complexes and modulate function of associated proteins. Mammalian genomes encode approximately 30 different members of this family and remotely related eukaryotic species also contain conserved tetraspanin homologs. Tetraspanins are involved in a number of fundamental processes such as regulation of cell migration, fusion, immunity and signaling. Moreover, they are implied in numerous pathological states including mental disorders, infectious diseases or cancer. Despite the great interest in tetraspanins, the structural and biochemical basis of their activity is still largely unknown. A major bottleneck lies in the difficulty of obtaining stable and homogeneous protein samples in large quantities. Here we report expression screening of 15 members of the human tetraspanin superfamily and successful protocols for the production in *S*. *cerevisiae* of a subset of tetraspanins involved in human cancer development. We have demonstrated the subcellular localization of overexpressed tetraspanin-green fluorescent protein fusion proteins in *S*. *cerevisiae* and found that despite being mislocalized, the fusion proteins are not degraded. The recombinantly produced tetraspanins are dispersed within the endoplasmic reticulum membranes or localized in granule-like structures in yeast cells. The recombinantly produced tetraspanins can be extracted from the membrane fraction and purified with detergents or the poly (styrene-co-maleic acid) polymer technique for use in further biochemical or biophysical studies.

## Introduction

Tetraspanins are small, approximately 30 kDa, integral membrane proteins characterized by four membrane spanning helices and a major extracellular domain that serves as a family “fingerprint” with a highly conserved CCG motif in animals and a GCCK/RP motif in plants [1]. Tetraspanins are found in higher eukaryotes, there are 31 family members in humans, 17 in plants (*Arabidopsis*) and up to 3 in fungi. There are no reported homologs in yeast, bacteria and archaea.

Tetraspanins are mediators of numerous biological processes like notch activity, B cell activation and immunity as well as cell motility, adhesion, fusion and proliferation. They are involved in wound healing and sperm-egg fusion and serve as host receptors for viral infections [2–5]. Moreover, they extend their influence on other cells by involvement in exosome mediated intercellular communication [6]. There is a vast amount of evidence for tetraspanin involvement in cancer, especially in metastasis processes [3,4]. Although tetraspanins are widely expressed by both normal and tumor cells, very often their down- or up-regulation is strongly correlated with disease outcome. Tetraspanins act by forming complexes with numerous other proteins, depending on the specific tetraspanin. Some tetraspanins appear to function as metastasis suppressors, while others indicate increased metastatic potential.

This article describes expression screening of 15 human tetraspanin superfamily members in the eukaryotic host *Saccharomyces cerevisiae* as well as the recombinant expression, purification and analysis of subcellular localization of tetraspanins CD63, CO-029, TSPAN7, TSPAN12 and TSPAN18, described briefly below.

**CD63 is** one of the most widely studied tetraspanins and one of the first characterized proteins in this family. It serves as a marker for various biological functions, particularly tumor dissemination. Its expression inhibits invasion potential in colon carcinoma and lung adenocarcinoma among others [7],[8]. Moreover CD63 is also present in high levels in exosomes and is thus used for quantification of human exosomes in plasma of melanoma patients [9]. Recent studies also identify CD63 as a receptor required for cell proliferation in a mouse lung-cancer model [10]. **CO-029** is expressed in melanoma as well as gastric, colon, rectal, and pancreatic carcinomas but not (or significantly less) in most normal tissues, and is associated with poor prognosis [11,12]. It has been shown to inhibit the cell movement by deregulation of the cell-matrix and cell-cell adhesions in colorectal cancer [13]. There are reported cases of using anti-CO-029 antibodies to inhibit tumor cell proliferation thus implicating CO-029 as an interesting target for cancer therapeutics [14,15]. **TSPAN7** has been identified as a specific surface marker of T-cell acute lymphoblastic leukemia and neuroblastoma [16]. It has also been shown to be a marker and possible therapeutic target for desmoplastic small round-cell tumor [17]. Mutations in the TSPAN7 gene lead to a form of X-linked mental retardation [18]. **TSPAN12** is involved in vascular development of the retina and mutations in its gene may cause the eye disorder familial exudative vitreoretinopathy [19,20]. It is implicated to be a pro-tumorigenic agent due to the regulation of the ADAM10 protein [21,22], as well as a metastasis suppressor in mouse lungs [23]. **TSPAN18** is less well studied but has been reported as highly expressed in endothelial cells and possibly involved in regulation of calcium signaling [24]. It plays a role in antagonizing neural crest epithelial–mesenchymal transition (EMT) and cell migration by supporting cell adhesion via the Cad6B protein [25].

Functionally, a key property of tetraspanins is their ability to organize at several levels in the membrane. The first level commonly consists of tightly associated homo-oligomers [26]. These building blocks assemble into larger structures that include other membrane proteins to produce a so-called tetraspanin web or tetraspanin enriched micro domains (TEMs) [27,28]. There is a very large number of proteins reported to interact with tetraspanins, including integrins, claudins, immunoglobulin superfamily proteins, G-protein coupled receptors, proteoglycans, proteases, cadherins and also soluble proteins involved in signaling such as phosphatidylinositol 4-kinase (PI4K) and Shc [26]. The formation of TEMs is also influenced by tetraspanin post-translational modifications and the presence of divalent cations e.g. Ca^2+^ and Mg^2+^ [29].

**Fig 1:**
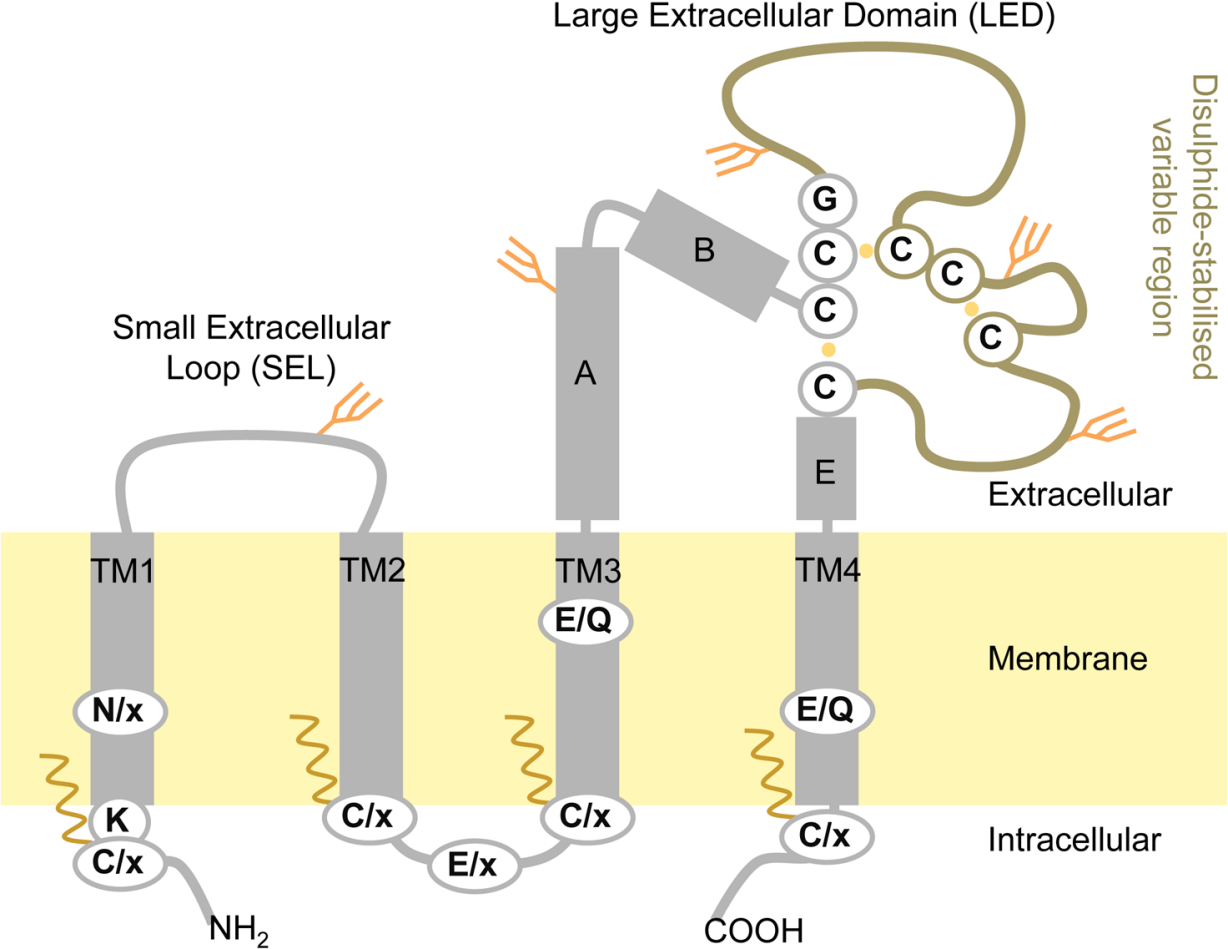
Tetraspanin topology scheme. Transmembrane helices are numbered 1–4, conserved helices in the large extracellular domain indicated with letters A,B,E according to nomenclature by Seigneuret et al. **[32]**. Conserved residues are shown in circles, where x stands for any amino acid. Possible post-translational modifications are indicated as palmitoylation sites shown as waves close to the intracellular side of the protein and available N-linked glycosylation sites shown as forks on the extracellular domains.

Tetraspanins are predicted to have an overall structure of a tight, rod-like shape [30,31], see Fig 1. The large extracellular domain (LED) varies in sequence and size, from 6 to 17 kDa, between homologs. The variable region of LED (Fig 1) contains the conserved CCG motif with stabilizing disulfide bonds in the extracellular domain [32–35]. The small extracellular loop of roughly 1 kDa displays much less variation. Both the N- and C-termini of tetraspanins are located in the cytoplasm and are usually as short as 5–15 amino acids. Some proteins in the family are however predicted to have longer termini tails of about 5–10 kDa. Tetraspanins are also known to be posttranslationally modified by N-linked glycosylation and/or palmitoylation, see Fig 1 [36,37]. The location of the tetraspanins in the cell membrane with key interacting domains on the extracellular side makes them amenable to manipulation by macromolecular drugs, such as monoclonal antibodies, and potentially useful as tumor biomarkers and therapeutic targets [4].

To date there is no high-resolution structure of a full-length tetraspanin. Detailed structural information has been obtained only for the human CD81 LED at 1.6 Å resolution [34,35]. The most successful purified full-length tetraspanin preparation reported to date is of the mouse Uroplakin complex, consisting of tetraspanin Upk1A and Upk1B, obtained from natural source and examined with electron microscopy revealing the complex structure at a resolution of 6Å [31]. There have been several other attempts to extract tetraspanins from native sources, in particular tetraspanins abundantly present in retina (the RDS/ROM1 complex), as well as the human CD9 from platelets and human CD151 from placenta, see Table 1 [27,38,39]. Even though a very interesting biochemical characterization was done, neither of these isolation protocols (except the Uroplakins) yield the protein amounts needed for structural studies. Higher amounts have been obtained for human CD81 when recombinantly produced using *Pichia pastoris*, yielding large amounts of a detergent solubilized full-length tetraspanin protein [40]. These studies have focused on individual tetraspanins. Here and in a previous study [41], we have adopted an expression-screening approach to investigate which human tetraspanin superfamily members can be expressed in *Escherichia coli* and *S*. *cerevisiae*.

**Table 1:** Experimental studies reporting tetraspanin production or isolation. MDCK, Madin-Darby Canine Kidney Epithelial Cells; HEK293, Human Embryonic Kidney 293 cells

**Full-length tetraspanins isolated from native source**				
***Tetraspanin***	***Origin***	***Source***	***Tag***	***Ref*.**
Uroplakin 1a/1b complex	Mouse	Urothelial plaques	n/a	[31]
RDS/ROM1 complex	Bovine	Retina	n/a	[27]
RDS	Bovine	Retina	n/a	[27]
CD9	Human	Platelets	n/a	[38]
CD151	Human	Placenta	n/a	[39]
**Recombinantly produced full-length tetraspanins**				
***Tetraspanin***	***Origin***	***Exp*. *System***	***Tag***	***Ref*.**
RDS	Human	MDCK	His_6_	[42]
CD81	Human	HEK293	Rho-1D4	[43]
	Human	*P*. *pastoris*	His_6_	[40]
Uroplakin 1b	Human	*E*. *coli*	GFP-His_8_	[41]
CD151	Human	*E*. *coli*	GFP-His_8_	[41]
TM4SF1^a^	Human	*E*. *coli*	GFP-His_8_	[41]
CD63	Human	*E*. *coli*	GFP-His_8_	[41]
	Human	*S*. *cerevisiae*	GFP-His_8_	This work
CO-029	Human	*S*. *cerevisiae*	GFP-His_8_	This work
TSPAN7	Human	*S*. *cerevisiae*	GFP-His_8_	This work
TSPAN12	Human	*S*. *cerevisiae*	GFP-His_8_	This work
TSPAN18	Human	*S*. *cerevisiae*	GFP-His_8_	This work

^a^ A tetraspanin superfamily member lacking the CCG motif [44].

Obtaining large quantities of tetraspanin protein is crucial for structural studies, generation of conformation specific antibodies as well as further biochemical and biophysical characterization. However, large-scale heterologous production of human membrane proteins is typically very challenging and often requires the use of an eukaryotic host [45]. Membrane protein overexpression in a eukaryotic host also raises the question of subcellular targeting, in what membrane(s) the proteins end up, and how the localization may influence the possibility to recover a protein sample suitable for further studies. The identification of well-expressing, properly folded and stable variants usually requires screening of several homologs, orthologs or mutational variants of a protein. Drew et al. introduced the green fluorescent protein (GFP)-based optimization scheme for membrane proteins produced in *S*. *cerevisiae* [46,47], which substantially simplified the screening and optimization of constructs, and has already been proved efficient in several cases [48].

Styrene maleic acid lipid particles (SMALPs) have been successfully used for isolation of intact membrane proteins in a preserved lipid environment. Among the first membrane proteins purified with this new extraction technique were bacteriorhodopsin and outer membrane protein PagP [49,50]. Recently this technique has received more attention and has also been applied to purification of G-protein coupled receptors [51,52], tyrosine kinase [53], transporters [54,55] and ion channels [56]. SMALPs have also been successfully used in isolation of protein complexes, in particular the respiratory complex IV [57] and bacterial reaction center [58]. For several of the above-mentioned proteins higher protein activity and/or stability in SMALPs compared to detergent solubilized material have been reported. The SMA polymer itself was previously also applied in medical research as drug carrier conjugates in the form of styrene maleic acid neocarzinostatin (SMANCS) [59,60]. The hydrolyzed form of the poly(styrene-co-maleic anhydride) polymer (SMA) is an amphiphilic polymer with alternating styrene/maleic acid moieties which can wrap around a fairly well defined size of lipid bilayer, creating approximately 12 nm-sized SMALP nanodiscs [58]. It auto-assembles directly within the membrane and thus allows protein solubilization and purification without the need of detergents [50]. Once encapsulated, the lipid-protein-polymer entities are very stable but pH sensitive. The SMALPs are characterized by a number of further advantages including biocompatibility, lack of interfering components and simple up-scaling. It has long been observed that once exposed to detergents membrane proteins may lose their functionality, hence SMA polymers appear to be a promising alternative extraction technique.

In this work we describe an efficient protocol for heterologous expression and extraction screening applied to 15 human tetraspanin superfamily members in yeast. We report expression and purification procedures with the use of detergents and the recently developed SMALP technique for protein extraction. We further study the subcellular localization of heterologously overexpressed human tetraspanin-GFP fusion proteins in *S*. *cerevisiae*. The same subset of human tetraspanins were also used in a previous study of overexpression in *E*. *coli* [41] allowing a direct comparison between the eukaryotic and prokaryotic expression hosts. Interestingly we found that the recombinant expression of human tetraspanins vary drastically between these two systems. The presented results and methods should also benefit further structural and biochemical studies of tetraspanins and their significance as therapeutic and diagnostic targets.

## Materials and Methods

### Cloning

Full-length genes of 15 human tetraspanins, listed in Table 2, were amplified from plasmids from the previous study of tetraspanin expression in *E*. *coli* [41]. No codon optimization was applied thus the original human DNA sequence is present in all constructs for each ORF used. DNA was amplified by polymerase chain reaction (PCR) using the Phusion high-fidelity DNA polymerase kit (Finnzymes) according to the manufacturer’s protocol. Forward primers were designed to include a 5′- TCGACGGATTCTAGAACTAGTGGATCCCCC -3′ overhang sequence preceding the ATG start codon and reverse primers included 5′-GCGCGCGGTACCCAC-3′ sequence to allow for homologous recombination in yeast. A yeast-enhanced GFP (yEGFP) [61] gene with a C-terminal octa-His tag and an upstream tobacco etch virus (TEV) protease site had previously been cloned into a pRS424GAL1 vector [62] giving the pRS424GAL1-GFP vector. A SmaI site had also been introduced preceding the TEVp-GFP-His8 sequence. The pRS426GAL1-GFP vector was linearized using the SmaI restriction enzyme prior to transformation. Transformation of the yeast strain FGY217 (MATα, *ura3-52*, *lys2Δ201*, *pep4Δ*) cells with the linearized pRS424GAL1-GFP vector and the PCR-products was performed according to previously published protocol [46]. Plates with-URA as a selection marker were incubated for 3 days at 30°C for colonies to appear. Transformants harboring homologously recombined plasmids encoding a tetraspanin-GFP fusion protein were used in further studies.

**Table 2:** Human tetraspanin superfamily members used for expression screening in *S*. *cerevisiae*.

Name	Alternative name
TSPAN3	TM4-A; TM4SF-8
TSPAN6	T245; TM4SF6; TSPAN6
TSPAN7	A15; MXS1; CD231; MRX58; CCG-B7; TM4SF2
TSPAN8	CO-029; TM4SF3
TSPAN9	NET-5; PP1057
TSPAN12	NET-2; TM4SF12
TSPAN18	-
Uroplakin 1b	UPIB; UPK1; TSPAN20
Uroplakin 1a	UP1A; UPIA; UPKA; TSPAN21; MGC14388
CD151 molecule	GP27; MER2; RAPH; SFA1; PETA-3; TSPAN24
CD81 molecule	S5.7; TAPA1; TSPAN28
CD9 molecule	5H9; BA2; P24; GIG2; MIC3; MRP-1; BTCC-1
CD63 molecule	MLA1; ME491; LAMP-3; OMA81H; TSPAN30
TSPAN31	SAS
TM4SF1^a^	L6; H-L6; M3S1; TAAL6

^a^ tetraspanin superfamily member lacking the CCG motif [44].

### Protein Expression Screening

Isolated colonies were inoculated from plates to 10 ml of SC-URA medium (2.0 g/l yeast synthetic drop-out medium without URA, 6.7 g/l yeast nitrogen base without amino acids) with 2% glucose in 50 ml falcon tubes. Cultures were incubated overnight in an orbital shaker at 280 rpm at 30°C. Overnight cultures were diluted to an OD_600_ of approximately 0.1 in 10 ml SC-URA medium with 0.1% glucose. After reaching an OD_600_ of 0.6, expression of tetraspanins was induced by addition of galactose solution to a final concentration of 2% and allowed to grow further. We found that induction for 22–24h produced the highest yield of expressed protein, with no improvement at longer induction times. After 22 hours of induction, cells were harvested by centrifugation at 3000g for 5 min. The supernatant was removed and cells resuspended in 200 μl of YSB (50 mM Tris-HCl pH 7.6, 5 mM EDTA, 10% glycerol, 1 mM PMSF). The cell suspension of 200 μl was transferred to a Nunc MicroWell 96-Well Optical-Bottom plate with black walls (Thermo scientific). GFP fluorescence emission was measured at 512 nm by excitation at 488 nm using Infinite M200PRO plate reader (Tecan). Glass beads were added to a final volume of 500 μl and an additional 500 μl YSB added, next cells were broken with a mixer-mill disruptor. Unbroken cells were removed by centrifugation at high speed in a desktop centrifuge for 5 min. The supernatant was transferred to a new tube, and 500 μl YSB added to the mixture of unbroken cells and glass beads. Cell breaking was repeated with the mixer-mill disruptor, unbroken cells removed by centrifugation and the supernatant collected. Crude membranes were collected by centrifugation at 18,000g for 1 h at 4°C in a desktop centrifuge (Beckman). The supernatant was discarded and the crude membranes resuspended in 200 μl YSB. GFP fluorescence emission was measured at 512 nm by excitation at 488 nm. Constructs yielding heterologous protein expression with detectable fluorescence in crude membranes were used in further analysis as described below.

### Expression Analysis

Clones producing the tetraspanin-GFP fusion proteins were used for further optimization of growth condition to yield higher amounts of recombinant protein. Colonies were inoculated from plates to 10 ml SC-URA medium with 2% glucose and incubated overnight at 30°C with shaking. Overnight cultures were diluted to OD_600_ 0.1 in 2 ml SC-URA medium supplemented with 0.1% glucose in 24-well plates in replicates of three. At OD_600_ = 0.6, protein production was induced with galactose added to a final concentration of 2%. The additives, DMSO and histidine were added to a final concentration of 2.5% and 40 μg/ml respectively. The optical density was measured and cells were harvested after 22 h of induction by centrifugation at 4,000g for 10 min. Cell pellets were resuspended thoroughly in 200 μl YSB, and then a 200 μl samples were taken and the GFP fluorescence was measured as described above.

### SDS-PAGE and In-gel-Fluorescence

For in-gel fluorescence measurements, samples were mixed with 2x loading buffer (50 mM Tris-HCl pH 7.6, 5% glycerol, 5 mM EDTA, 0.02% bromophenol blue, 4% SDS, 0.05 M DTT) and incubated at room temperature for 10 min. A 5–20 μl of sample mix was loaded on 14% Tris-glycine gels (Invitrogen) and run at 125 V for 90 min with MES-buffer. Care was taken to not overheat the gel, as that results in GFP unfolding. In-gel GFP fluorescence was immediately visualized with a LAS-1000 charge-coupled device (CCD) imaging system (Fujifilm).

### Glycosylation test with endoglycosidase H

To investigate glycosylation status of heterologously expressed human tetraspanins in *S*. *cerevisiae*, a sample of crude isolated yeast membranes containing tetraspanin-GFP fusion proteins were treated with endoglycosidase H (EndoH). Yeast membranes were mixed with EndoH (Roche) and 9x Buffer (0.25 M sodium citrate) and thereafter incubated for 2 h at 37°C. Samples were then loaded on SDS-PAGE, as described above, and investigated using in-gel fluorescence.

### Confocal microscopy

For confocal microscopy experiments *S*. *cerevisiae* cells expressing tetraspanin proteins were grown and induced with the same conditions and time-scale as for protein production screening. A suspension of 1–2 ml yeast culture was pelleted by centrifugation at 2000g. Cells were washed three times with PBS (100 mM sodium phosphate buffer pH 7.2, 150 mM NaCl) and incubated in the dark for 30 min with an FM4-64FX dye (Life Technologies, UK), followed by 45 min incubation with concanavalin A 647 (Life Technologies, UK), and 1 hour of incubation with ER-Tracker Blue-White DPX (Life Technologies, UK) according to the manufacturer’s protocol. The cells were washed three times with PBS after incubation with each dye. Next, cells were suspended in PBS and mildly (to preserve the native GFP fluorescence) fixed for 1 h in dark with 1% final concentration of formaldehyde in PBS supplemented with 1 mM MgCl_2_ to stabilize cell morphology and architecture. All incubation and fixation procedures were done with gentle mixing in dark at 4°C. After fixation, cells were washed again three times in PBS and a small fraction of the cell pellet was suspended in a Fluorescence mounting medium (Dako, Agilent, USA). An aliquot of 5 μl was immediately placed on microscope slide and overlaid with a cover slip, then left overnight in dark at 4°C to allow cell immobilization. Images were taken using a Zeiss LSM710 confocal microscope.

### Detergent solubilization

Membranes were isolated from up-scale growths done with a LEX bubbling system (Harbinger Biotech). The LEX system allows large cell culture volumes by bubbling air through the growth media, which provides both good aeration as well as stirring. Cells were harvested and washed with buffer containing 5% glycerol, 50 mM Tris-HCl pH 7.6 and pellets stored at -80°C. Frozen cell pellets were thawed on ice, resuspended in YSB and passed three times through an Emulsiflex C3 (Avestin) cell disruptor at 25 kPsi. Unbroken cells and cell debris were removed by centrifugation at 15,000g for 20 min. Membranes were collected by ultracentrifugation at 180,000g for 45 min, then resuspended and homogenized in MRB buffer (50 mM Tris-HCl pH 7.8 and 150 mM NaCl).

As a starting point for detergent screening four detergents (Anatrace, USA) with diverse chemical properties (Table 3) representing two detergent classes were selected. A nonionic detergent n-Dodecyl-B-D-Maltopyranoside (DDM), being a “standard” class of its own as it is one of the most commonly used compounds in membrane protein purification. A 2,2-didecylpropane-1,3-bis-β-D-maltopyranoside (LMNG), another nonionic detergent with branched-chains, which is reported to form more stable protein-detergent complex and stabilizes the protein itself [63]. From the second zwitterionic class, Fos-Choline-12 (FC12), with strong extraction capabilities and n-Dodecyl-N,N-Dimethylamine-N-Oxide (LDAO) as one of the most successful of the zwitterionic detergents [64] were selected, see Table 3. Solubilization samples of 1 ml containing solubilization buffer (50 mM Tris-HCl pH 7.6, 150mM NaCl, 5% glycerol), 1% (w/v) detergent and isolated membrane suspension (with a total protein concentration of approximately 5 mg/ml) were used. Protein concentration in the membrane suspension was determined using the DC protein assay (BioRad) according to the manufacturer’s protocol. Membranes were solubilized for 1 h with slow agitation at 4°C, and unsolubilized material removed by ultracentrifugation at 175,000g for 30 min. The GFP fluorescence was measured on 100 μl samples taken before and after ultracentrifugation from the solubilization mixture. The degree of solubilization was determined by comparison of these two values.

**Table 3:** Detergents and hydrolyzed forms of SMA polymers used in screening for solubilization efficiency of membranes containing TSPAN-GFP fusion proteins. Listed are also the final concentration used for solubilization.

Detergent / Polymer	Abbreviation	Conc. (%)
n-Dodecyl-B-D-Maltopyranoside	DDM	1
n-Dodecyl-N,N-Dimethylamine-N-Oxide	LDAO	1
Fos-Choline-12	FC12	1
2,2-didecylpropane-1,3-bis-β-D-maltopyranoside	LMNG	1
Poly(Styrene-co-Maleic Anhydride) 3000	SMA-3000	2.5
Poly(Styrene-co-Maleic Anhydride) 2625	SMA-2625	2.5
Poly(Styrene-co-Maleic Anhydride) EF30	SMA-EF30	2.5

### Detergent-free solubilization

A recently-developed technique based on hydrolyzed form of poly(styrene-co-maleic anhydride) polymer (SMA) forming in contact with membrane the lipid nanoparticles (SMALP) that contain membrane proteins together with native lipids was applied as alternative solubilization method. Based on the literature indicating that low molecular weight SMA polymers with 3:1 styrene to maleic acid molar ratio are suitable for membrane protein isolation [65] three polymers were selected for tests. A SMA-3000 copolymer with approx. 3:1 styrene to maleic anhydride molar ratio and MW 9.5 kDa. A very similar SMA EF30 with about 3:1 molar ratio and MW 9.5 kDa. And a SMA 2625 that is a partial monoester of a SMA resin with MW 9.0 kDa (Cray Valley, USA), see Table 3. A 10% suspension of SMA resin was hydrolyzed by 4 h refluxing in 1 M NaOH at 80°C and allowed to cool to room temperature. The hydrolyzed polymer was recovered by precipitation in acidic conditions [66], separated by centrifugation and redissolved in Tris buffer pH 8.0. The resulting solution was dialyzed overnight against solubilization buffer at 4°C then passed through a 22 μm filter and stored as 5% stock solution at 4°C. The same membrane protein solubilization procedure as described above was used except that 2.5% final concentration of hydrolyzed SMA solution was used instead of the detergent.

### Up-scale production and purification of CO-029

CO-029-GFP was selected for a large-scale expression and purification. Starter cultures were grown in 250 ml SC-URA media with 2% glucose overnight at 30°C, in flasks with 280 rpm shaking. Overnight cultures were diluted to an approximate OD_600_ of 0.1 in 1.4 l SC-URA media containing 0.1% glucose in 2-liter flasks. 150 μl of Antifoam 204 (Sigma) was added to 1.4 l of media to prevent foaming. Over 11 liters of growth media split to eight 2 liter flasks were used in the LEX-large scale bubbling system set at 30°C and vigorous aeration. Induction was initiated by addition of 150 ml of 20% galactose (final concentration 2%) and 30 ml DMSO (final conc. 2%) to each flask when the growth culture reached an OD_600_ of 0.6. Cells were harvested by centrifugation at 12,000g for 15 min after 20 h of induction. Cell pellets were washed with buffer containing 5% glycerol, 50 mM Tris-HCl pH 7.6 and stored at -80°C for further use. Frozen cell pellets were thawed on ice and resuspended in YSB, homogenized and passed four times through an Emulsiflex C3 (Avestin) cell disruptor at 25 kPsi. Unbroken cells and cell debris were removed by centrifugation at 15,000 g for 20 min. Membranes were then collected by ultracentrifugation at 180,000g for 45 min, and the pellet resuspended and homogenized in MRB. Total protein concentration in the membrane suspension was determined using the DC protein assay (BioRad) according to the manufacturer’s protocol.

Membranes containing CO-029-GFP fusion protein were solubilized using 1% Fos-Choline12 detergent (Anatrace, USA) in MRB buffer at 4°C for 1 h 30 min. Unsolubilized material was removed by ultracentrifugation at 150,000g for 45 min. Supernatant containing solubilized material supplemented with 10 mM imidazole was incubated by tumbling overnight at 4°C with Ni-NTA agarose resin (Qiagen), 1 ml resin was used per 1 mg of CO-029-GFP fusion protein as estimated by GFP fluorescence.

The resin with bound protein was collected on a plastic gravity flow column and the flow through discarded. The resin was washed and the protein was eluted with an increasing imidazole concentration in buffer containing 50 mM Tris-HCl pH 7.8, 150 mM NaCl and 0.1% DDM. Briefly, 20 column volumes of 10 mM imidazole, followed with 50 mM imidazole were used for wash. Protein was eluted with 300 mM imidazole in the same buffer in 2 ml fractions. GFP fluorescence was monitored at each step.

His-tagged TEV protease was added at a TEV:CO-029-GFP molar ratio of 2:1. Glutathione and oxidized glutathione was added to a final concentration of 3 mM and 0.3 mM respectively and the mixture incubated at 4°C overnight. The cleavage mixture was then applied to a HiPrep 26/10 desalting column (GE LifeSciences) pre-equilibrated with 20 mM Tris-HCl pH 7.8, 150 mM NaCl, 0.1% DDM. Protein-containing fractions were pooled and Ni-NTA agarose resin added for removal of the TEV protease and the cleaved-off GFP. The mixture was incubated by tumbling at 4°C for 1 h, transferred to a gravity flow column and the flow through collected. The CO-029 sample was concentrated using a 100 kDa cut-off filter. Purified protein samples were applied to an analytical Superdex 200 5/150 (GE Life Sciences) gel filtration column equilibrated with 20 mM Tris-HCl pH 7.6, 150 mM NaCl and 0.03% DDM buffer at 0.25 ml/min flow rate and the elution profile monitored by absorbance at a wavelength of 280 nm.

## Results

### Human tetraspanins can be produced in *S*. *cerevisiae*

**Fig 2:**
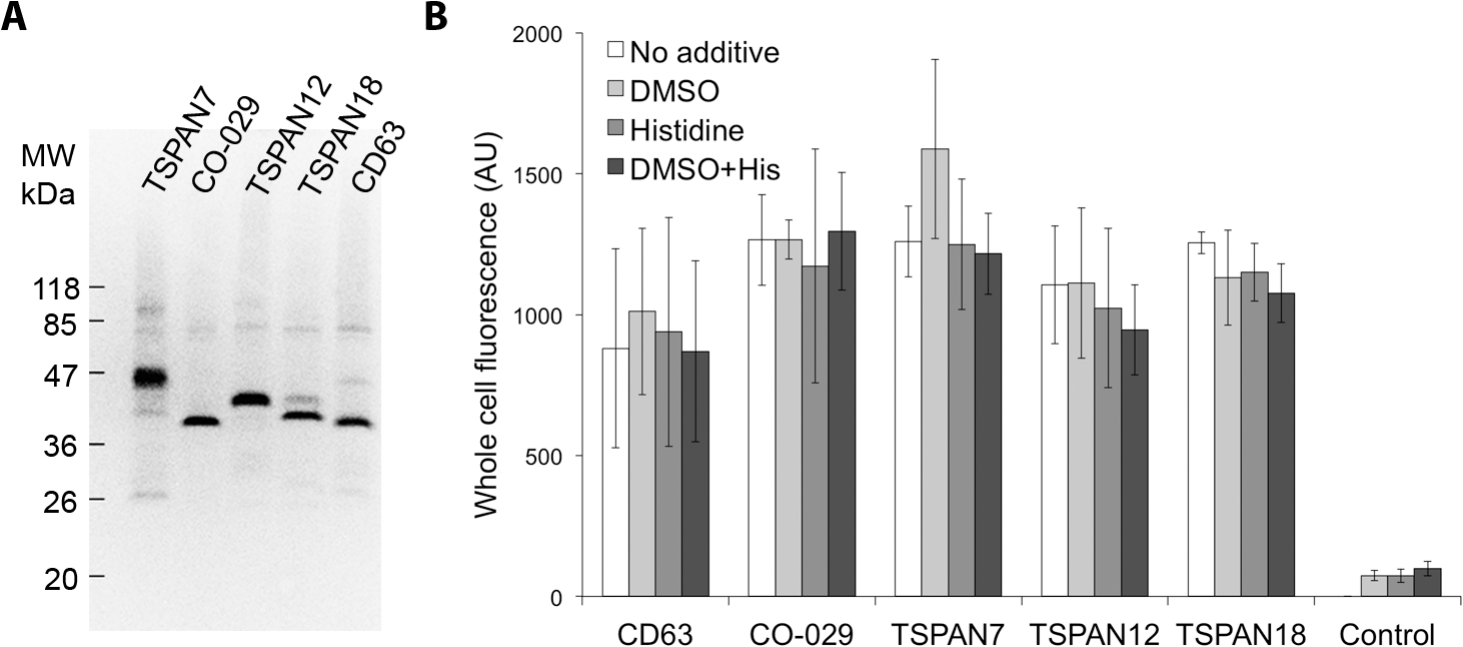
Recombinant expression of tetraspanins in *S*. *cerevisiae*. **(A)** SDS/PAGE in-gel fluorescence of crude membranes isolated from *S*. *cerevisiae* expressing GFP fusion of TSPAN7, CO-029, TSPAN12, TSPAN18 and CD63. **(B)** Quantification of whole cell fluorescence of TSPAN-GFP fusion proteins during induction supplemented with either 2.5% DMSO or 0.04% histidine, or both.

Here we report heterologous expression screening of 15 human tetraspanin superfamily members as GFP-fusion constructs produced in *S*. *cerevisiae*. Five of the examined constructs resulted in successful production of membrane-inserted tetraspanins. The other 10 constructs show no, by fluorescence measurement, detectable protein production. The protein expression and membrane insertion was confirmed by SDS-PAGE in-gel fluorescence of isolated membranes for CD63, CO-29, TSPAN7, TSPAN12 and TSPAN18, see Fig 2A. As commonly seen for membrane-proteins [67,68] and previously observed for tetraspanins produced in *E*. *coli* [41], membrane protein GFP fusion proteins may migrate with lower than expected apparent molecular mass in SDS-PAGE gels. It was recently reported that at certain acrylamide concentration (referred to as lower than equivalent gel mobility) the increased net charge of membrane proteins dominate causing higher than expected apparent protein mobility. The same study shows that the interplay between gel density and membrane protein net charge, as well as its effective molecular size, has a “switch point” on the direction (slower/faster) and level of the abnormal mobility of helical membrane proteins in SDS-PAGE [69] which could explain part of the gel-shift observations in the tetraspanins case. Still, in case of four tetraspanins (CD63, CO-029, TSPAN12 and TSPAN18) the relative band migration on the SDS-PAGE gel correlates with the relative calculated molecular mass. TSPAN7-GFP behaves differently and migrates as a species larger than expected and also appears as several clustered bands, which suggests posttranslational modification of this protein when expressed in *S*. *cerevisiae*.

The yield of membrane protein purification is often low and the amount of produced protein was therefore optimized with the use of growth media supplements of 2.5% DMSO or 0.04% histidine, or both, see Fig 2B, previously shown to improve the *S*. *cerevisiae* expression efficiency [46,70]. Non-induced cell cultures were used as a control. Neither of the tested conditions showed substantial improvement in expression level, nonetheless DMSO was used in the up-scale growth due to a slight preference in case of this additive.

### Subcellular localization and posttranslational modifications of recombinant tetraspanins

**Fig 3:**
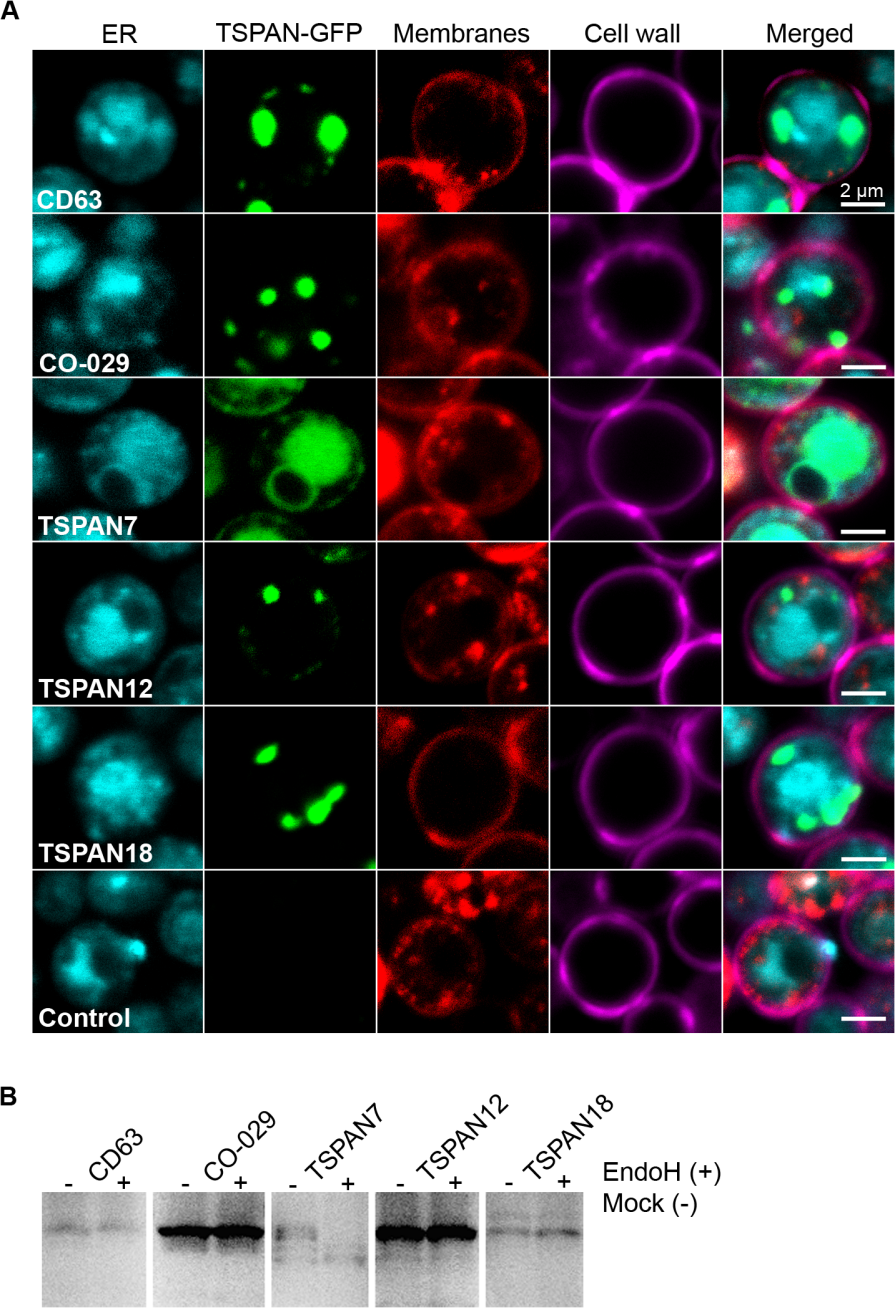
Cellular localization and posttranslational modification of recombinant human tetraspanins. **(A)** Representative confocal images of *S*. *cerevisiae* cells expressing human tetraspanin proteins with a C-terminal GFP tag. ER membranes were visualized by ER-tracker Blue-White DPX (cyan), tetraspanin-GFP fusion proteins were visualized by a native GFP fluorescence (green), membranes were visualized by FM 4-64FX (red) and cell wall was visualized by Concanavalin A Alexa Fluor 647 Conjugate (magenta). Each channel is shown separately for clarity and then merged. Scale bar: 2 μm **(B)** SDS-PAGE in-gel fluorescence of crude *S*. *cerevisiae* membranes containing GFP-tagged tetraspanins after EndoH treatment (+) and controls prepared in the same way but without EndoH (-). A shift of the fluorescent band to a lower molecular mass after EndoH treatment is observed only in case of TSPAN7.

The subcellular localization and targeting of human tetraspanins in yeast was investigated using confocal microscopy. Conveniently the GFP-tagged tetraspanins could be visualized without the use of additional antibodies or production of protoplasts. As seen in Fig 3A, the localization of the GFP-tetraspanin fusion proteins varies. TSPAN7-GFP is detected in the form of an outer ring in the vicinity of the cell membrane and a large, dispersed patch within the cell. This pattern overlays almost perfectly with an ER-tracker dye, confirming dispersion of this protein throughout the endoplasmic reticulum (ER). The confocal microscopy images of cells expressing the GFP-tagged tetraspanins CD63, CO-029, TSPAN12 and TSPAN18 show a vastly different pattern. The small granule-like patches of fluorescent protein are of various sizes and spread throughout the cell with some of them located in the vicinity of the plasma membrane or localized to the ER or other sub-cellular membranes, see Fig 3A.

TSPAN7-GFP revealed a slower than expected migration on SDS-PAGE and substantially different membrane localization than other tested tetraspanins. *S*. *cerevisiae* contains many mannose-rich glycoproteins and TSPAN7 has several predicted glycosylation sites. Therefore the possibility of posttranslational modifications was investigated by subjecting a crude membrane preparation containing TSPAN7-GFP to enzymatic treatment with EndoH glycosidase, which hydrolyzes oligosaccharides of glycoproteins. TSPAN7-GFP displays a band shift to a lower molecular mass in the EndoH-treated sample but not in the mock control. The other tested tetraspanins (CD63, CO-029, TSPAN12 and TSPAN18) did not change their apparent molecular mass after EndoH treatment, see Fig 3B. We conclude that TSPAN7 is indeed glycosylated when produced in *S*. *cerevisiae*.

### Comparison of detergent and detergent-free extraction

**Fig 4:**
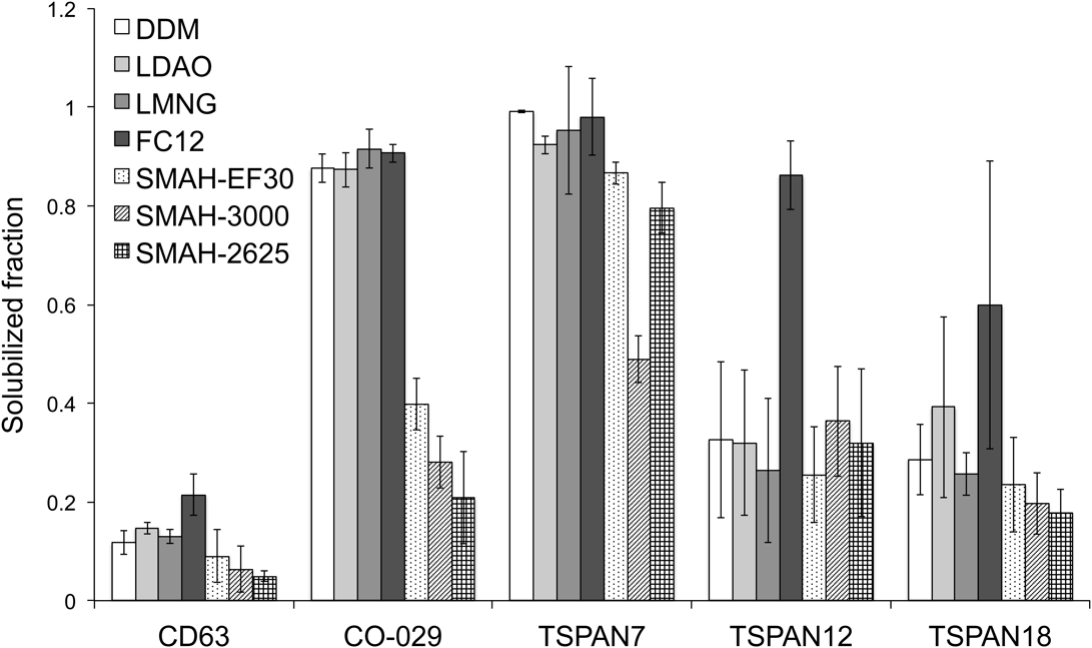
Solubilization efficiency of TSPAN-GFP fusion proteins. The solubilization degree of five tetraspanin-containing yeast membranes with the help of detergents and SMA polymers. The solubilization ratio was determined by comparing the fluorescence counts of solubilized material after ultracentrifugation to the mix before separation of solubilized and non-solubilized material.

Protein extraction screening was performed for all five tetraspanin constructs employed in expression analysis using both the detergent and SMALP approach. As seen in Fig 4 the solubilization of membranes containing tetraspanins varies greatly, not only between the different constructs, but also between the different detergents and SMALPs. The solubilization of membranes containing CD63 yielded no CD63-GFP in the soluble fraction after ultracentrifugation independent of the agent used. The zwitterionic Fos-Choline 12 was efficient in protein extraction from yeast membranes, for all other tetraspanins tested. Any other agent did not solubilize TSPAN12-GFP and TSPAN18-GFP to a satisfactory degree. Both CO-029-GFP and TSPAN7-GFP were solubilized in all detergents tested. TSPAN7-GFP was also extracted using SMALPs, which interestingly was not as successful for CO-029-GFP, see Fig 4. In our test the hydrolyzed form of SMA EF30, used to create SMALPs, was more efficient in extraction of tetraspanins from yeast membranes than SMA3000 and SMA2625. Altogether, detergent-free protein extraction with SMALP was of similar efficiency compared to detergents in most cases. Extraction of CO-029 was significantly more efficient with detergents compared to SMALP.

### Up-Scale Purification of CO-029

**Fig 5:**
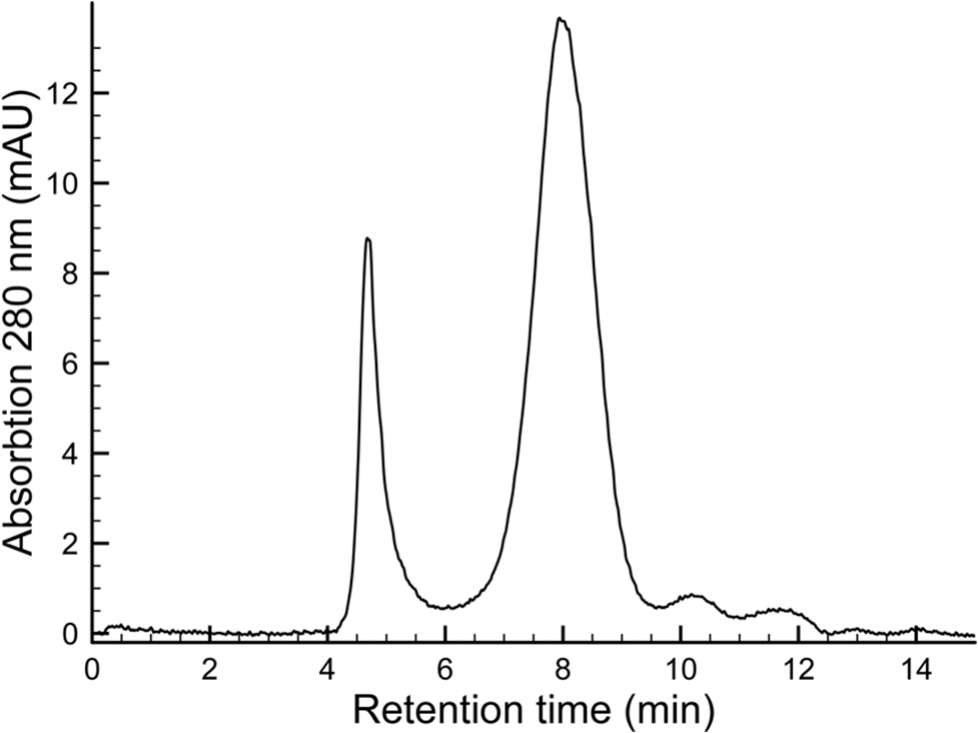
Size exclusion chromatogram of purified CO-029. Size exclusion of the purified CO-029 protein after proteolytic cleavage and removal of the GFP-tag. The elution was monitored with the absorbance at 280 nm.

As a case study for the tetraspanin protein family, we performed an up-scale growth, extraction and purification of CO-029 for use in further biochemical and biophysical studies. The His-tagged tetraspanin-GFP fusion protein was successfully solubilized from yeast membranes using Fos-Choline 12. As this detergent is known to be harsh and commonly unsuitable for other studies, it was exchanged for the milder n-Dodecyl-β-D-Maltopyranoside (DDM) during subsequent purification steps. The GFP tag was successfully removed after an initial immobilized metal ion affinity chromatography (IMAC) purification step by incubation with TEV-protease followed by reverse IMAC purification. The proteolytically cleaved and further purified CO-029 sample was thereafter applied to an analytical size exclusion column for analysis. The gel filtration elution profile, monitored at 280 nm absorbance, appeared as a single symmetric peak at approximately 8 min, indicating a monodisperse homogeneous sample with the expected size for the protein in DDM micelles, see Fig 5. In addition, a smaller void peak at approximately 4.5 min was observed, which corresponds to a fraction of aggregated protein.

## Discussion

In general, integral membrane proteins are assembled and achieve their final tertiary structure at the endoplasmic reticulum (ER) [71], and are thereafter transported to the Golgi apparatus and subsequently to the plasma membrane or degraded if failing the quality control [72,73]. Tetraspanins are reported to be targeted to and localized in the plasma membrane in mammalian cells. All extracted tetraspanins presented here were found in the isolated membrane fraction of the yeast cell lysate, see Fig 2. Our investigation of *in vivo* localization of full-length human tetraspanin-GFP fusion proteins expressed in *S*. *cerevisiae* showed that they are either distributed throughout the ER membrane (TSPAN7, Fig 3A) or form granule-like structures (CD63, CO-029, TSPAN12 and TSPAN18, Fig 3A). We have also performed size exclusion chromatography of CO-029 and observed a small fraction of the protein in aggregated form, while the major part eluted as a homogeneous monodisperse protein sample of expected size, as judged from the single peak after gel filtration, see Fig 5. We can thus conclude that the described tetraspanins are membrane inserted, and can be obtained as a homogenous sample. The use of GFP does not only visualize the protein of interest but also works as a quality control. It is presumed that the C-terminal GFP is correctly folded, and fluorescent, only upon integration of the upstream membrane protein into the membrane [47,74]. Although this is true in case of *E*. *coli* over-expression, in eukaryotic cells (like yeast used here) it has been shown that GFP may remain fluorescent even if the fusion protein is misfolded [47,75]. For this reason, for further studies correct folding should be verified using specific assays for each given protein. Some tetraspanins require a partner protein to successfully leave the ER and to be further targeted to plasma membrane [76] and therefore it cannot be excluded that due to the lack of their natural partner proteins they are either retained in the ER, preserved in the Golgi apparatus or targeted to the vacuoles, which has previously been observed for integral membrane proteins [77].

**Fig 6:**
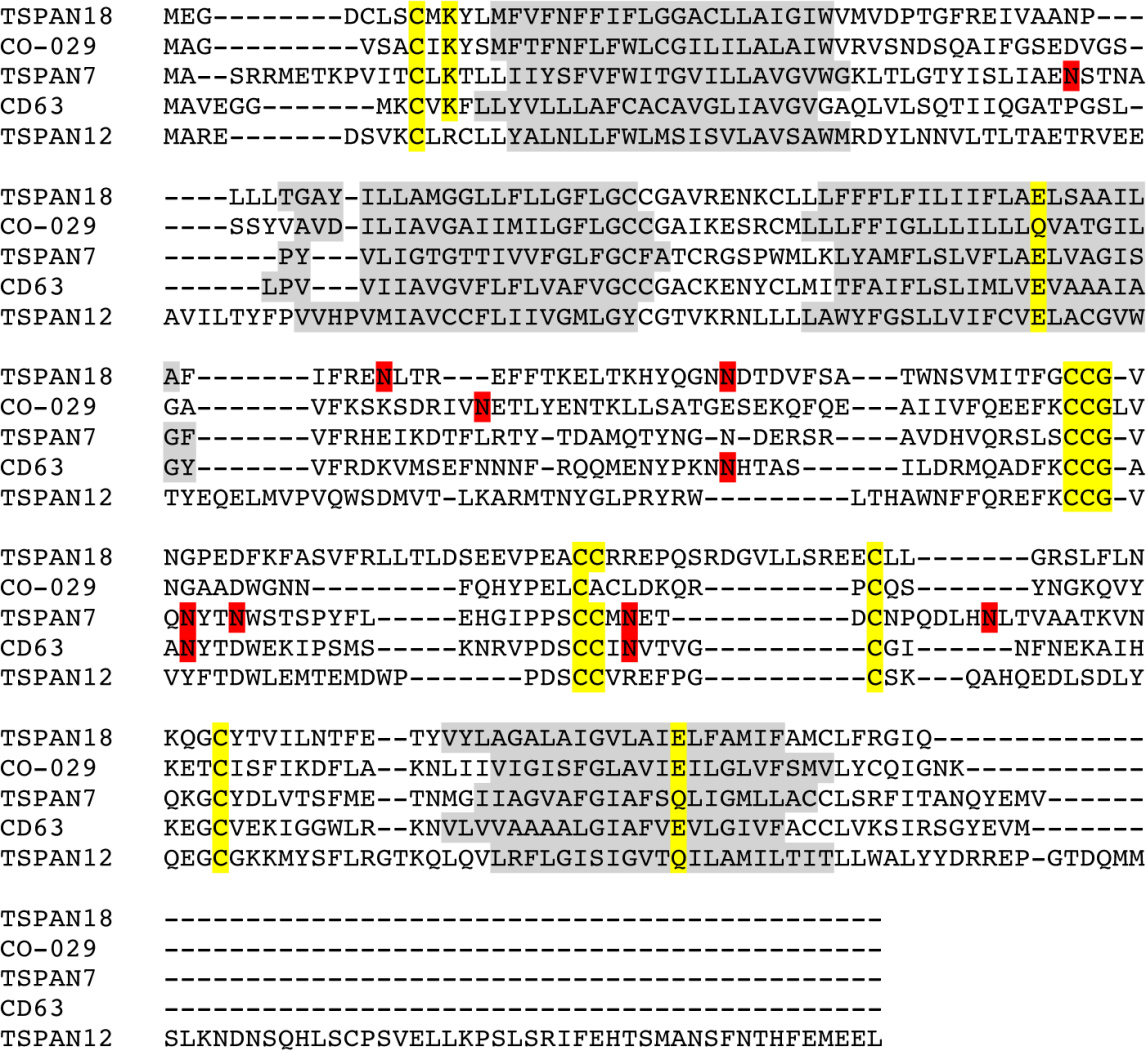
Sequence alignment of selected tetraspanins. Predicted localization of transmembrane helices with TOPCONS server [78] are indicated with a gray background. Residues conserved across the family–indicated in yellow and predicted glycosylation sites [79] are indicated with a red background. Sequence alignments were generated with the ClustalW server [80] by feeding 219 tetraspanin sequences from diverge spices to improve alignment statistics.

Many members of the tetraspanin family undergo post-translational modifications, commonly palmitoylation and glycosylation [36]. TSPAN7 has five predicted N-linked glycosylation sites, one on the SEL and 4 sites on the LED whereas TSPAN12 has no predicted N-glycosylation sites, as shown in Fig 1 and Fig 6. The post-translational machinery differs between yeast and humans, and this can cause alterations in the final modifications of recombinant proteins, also causing different sorting than in the native host. Moreover this could also be a limiting factor for obtaining expression of human proteins in yeast. It should be noted that heterologous overexpression of integral membrane proteins in yeast may also cause ER stress, which can become a limiting factor in protein production.

We have found no significant correlation between the heterologously produced protein localization and its solubility with detergents in this study. Protein extraction with SMALP did not give higher yields in comparison to detergent extraction, but comparable in most cases. In this context it should be noted that SMALPs seem to be milder and more protein-friendly in many cases [55] and thus a useful addition to detergent-based membrane protein solubilization screening.

There are only a few previously reported studies of isolated tetraspanins, purified from native source or recombinantly produced, as described in the introduction. The cryo-EM structure of Uroplakins at 6Å resolution [31] serves as the only experimental full-length tetraspanin model to date. There is also an in-silico model of full-length CD81 [30], based on the CD81 large extracellular domain crystal structure [34]. Exploring which tetraspanins can be produced in high amounts in *E*. *coli* [41] or *S*. *cerevisiae* (this work) lays the foundation for further structural and biochemical characterization of this medically very important superfamily.

Tetraspanin expression screening in *E*. *coli* [41] used the same starting set of proteins. Interestingly the *E*. *coli* study resulted in successful overexpression of a different subset of tetraspanin proteins compared to the expression in *S*. *cerevisiae* presented here. The best expressing tetraspanins in *E*. *coli*, UPK1B, CD151 and TM4SF1 were not possible to produce in yeast using the conditions tested within this study. The weakly expressed tetraspanin CD63 (five times less when compared to UPK1B) could also be produced in yeast but was not extractable from the yeast membranes.

## Conclusions

Human tetraspanins CO-029, TSPAN7 TSPAN12, TSPAN18 and CD63 can be produced by heterologous expression in *S*. *cerevisiae*. We have found them to be membrane-inserted and dispersed in the ER (TSPAN7) or present as, granule-like structures. We further showed that CO-029, TSPAN7, TSPAN12 and TSPAN18 could be extracted from isolated membranes using either detergents or SMALPs. Together, using *E*. *coli* and *S*. *cerevisiae* it is possible to produce and extract 7 of the 15 human tetraspanin superfamily members tested in quantities suitable for further structural, biochemical and biophysical studies.
